# A comparison of pediatric ocular injuries based on intention in patients admitted with trauma

**DOI:** 10.1186/s12886-018-1024-7

**Published:** 2019-01-29

**Authors:** Ryan Gise, Timothy Truong, Afshin Parsikia, Joyce N. Mbekeani

**Affiliations:** 1 Department of Ophthalmology & Visual Sciences, Montefiore Medical Center/Albert Einstein College of Medicine, 3332 Rochambeau Avenue, 3rd floor Ophthalmology Offices, Bronx, NY 10467 USA; 2 Montefiore Medical Center, Albert Einstein College of Medicine, 3332 Rochambeau Avenue, 3rd floor Ophthalmology Offices, Bronx, NY 10467 USA; 3 Department of Surgery (Trauma), Jacobi Medical Center, 1400 Pelham Parkway, Bronx, NY 10461 USA; 4 Department of Surgery (Ophthalmology), Jacobi Medical Center, 1400 Pelham Parkway, Bronx, NY 10461 USA

**Keywords:** Pediatric ocular trauma, Assault-related injuries, Self-inflicted trauma, Suicide, Unintentional trauma, National Trauma Data Bank (NTDB)

## Abstract

**Purpose:**

Pediatric ocular trauma is a major source of morbidity and blindness and the number of epidemiological studies is incommensurate with its significance. We sought to determine differences in epidemiologic patterns of pediatric ocular injuries based on intention.

**Methods:**

A retrospective review of the National Trauma Data Bank (2008–2014) was performed and patients < 21 years old, admitted with trauma and ocular injury, were identified using ICD-9CM codes. Demographic data, types of injury and external circumstances including intention were tabulated and analyzed with students’ t and chi-squared tests and logistic regression. Statistical significance was set at *p* < 0.05.

**Results:**

Fifty-eight thousand seven hundred sixty-five pediatric patients were admitted for trauma and ocular injuries. The mean(SD) age was 11.9(6.9) years. Most patients were male (68.7%) and White (59.1%). Unintentional injuries (76.3%) were mostly associated with falls (OR = 13.4, *p* < 0.001), assault (16.3%) with firearms (OR = 9.15, p < 0.001) and self-inflicted trauma (0.7%) also with firearms (OR = 44.66, *p* < 0.001). There was increasing mean(SD) age from unintentional, 12.9(6.6) years and assault 12.3(8.1) years to self-inflicted trauma, 17(3.4) years. The 0-3 year age group had highest odds of open adnexa wounds (OR = 30.45, *p* < 0.001) from unintentional trauma, and traumatic brain injury (TBI) (OR = 5.77, *p* < 0.001) and mortality (OR = 8.52, p < 0.001) from assault. The oldest 19-21 year group, had highest odds visual pathway injuries (OR = 8.34, p < 0.001) and TBI (OR = 1.54, *p* = 0.048) from self-inflicted trauma and mortality (OR = 2.08, p < 0.001) from unintentional trauma.

**Conclusion:**

Sight-threatening injuries were mostly associated with unintentional trauma in the youngest group and self-inflicted trauma in the oldest group. Patterns emerged of associations between demographic groups, mechanisms, types of injury and associated TBI with intention of trauma.

**Electronic supplementary material:**

The online version of this article (10.1186/s12886-018-1024-7) contains supplementary material, which is available to authorized users.

## Introduction

Pediatric trauma, from both accidental and intentional causes, is a leading cause of morbidity and mortality [1, 2]. A recent study found that approximately 1 in 10 pediatric patients admitted for trauma in the US were assault victims [3]. In 2014, an estimated 702,000 children were physically abused [4]. These patients had three times greater risks of mortality when they had severe head injury [3]. Given its anatomic location, the eye is at risk of damage in head trauma thus ocular trauma is a significant cause of morbidity in the pediatric population [5–7]. Visual impairment in childhood can have marked longterm effects on daily functioning and psychosocial development [8]. The incidence of ocular injury serious enough to result in hospiatlizaion ranges from 8.85 to 15.2 per 100,000 children [9, 10].

Some of the most concerning forms of ocular injuries are those attibutable to assault injuries in infants who are in an active stage of development. Epidemiology and investigations into diagnostic accuracy of ocular findings in Non-Accidental Trauma (NAT) remain active areas of research [11–13]. A study evaluating non-ophthalmologist accuracy in the diagnosis of retinal hemorrhages found that 13% were incorrectly documented as normal [14]. While retinal findings in NAT are widely reported, there are limited studies of other ophthalmic injuries resulting from assault [15]. Outside the ophthalmic literature, several studies have investivated the epidemiology of assault in children, comparing resulting injuries to unintentional trauma, to identify disparate patterns [1, 3, 16–19]. In this study, we used a large national database to evaluate pediatric ophthalmic injuries in patients admitted for trauma in order to elucidate if specific patterns of injuries based on intention exist.

## Methods

### Data collection

This study is a retrospective review of the National Trauma Data Bank (NTDB) from 2008 through 2014. It was reviewed and approved by the Institutional Review Board at Montefiore Medical Center/Albert Einstein College of Medicine. The NTDB is a data bank established and maintained by the American College of Surgeons [20]. It was founded with the goals of improving the care of patients injured by trauma through epidemiological research. Over 900 centers contribute de-identified patient data voluntarily and patients meet inclusion criteria if they are either admitted to the hospital for traumatic injury or expire from injuries while in the Emergency Department. Each patient must and have been diagnosed with International Classification of Diseases, 9th Revision, Clinical Modification (ICD-9CM) code between 800.00 and 959.9. The methods of our data collection and statistical analysis have been published in a previous publication and are summarized below [21, 22].

All patients under 21 years of age, admitted for trauma with concomitant ocular injury were identified using ICD-9CM codes. The patients were subdivided by age into groups for comparative analysis. Age group brackets were chosen based on developmental milestones (0-3 years, 4-6 years, 7-11 years, 12-18 years and 19-21 years) [23, 24]. Included ocular injuries were grouped depending on anatomic location. The Center for Disease Control (CDC) criteria for traumatic brain injury (TBI) was used to make this diagnosis [25, 26]. The patients were further separated into groups based upon the classification of the intent of the injury they suffered - either unintentional, assault or self-inflicted.

For all included patients, we collected demographic information as well as characteristics of their injuries including the type, mechanism and intent of injury and whether the injury was blunt or penetrating. Additional patient information tabulated included the Glasgow Coma Score (GCS) (from the Emergency Department and Emergency Medical Services), Injury Severity Score (ISS), length of admission and mortality. ISS is a designation that is used to quantify the severity and extent of trauma and ranges from 0 to 75 [27]. It is calculated using assigned numerical values for injuries depending on their bodily location and severity. Higher trauma severity is indicated by higher scores and scores over 15 are classified as major trauma [27].

### Statistical analysis

For continuous variables the mean, median, standard deviation (SD) and interquartile range (IQR) were calculated. Student’s t-test, chi-squared test, univariate and multivariate logistic regression analysis were utilized to determine association between different variables. Odds ratio (OR) and confidence intervals (CI) were used to express relative risk. All statistical calculations were performed using SPSS software (Statistical Package for Social Science, IBM Corp, Armonk, NY) and all graphs and charts were generated in and Microsoft excel (Microsoft Corp, Redmond, WA). Patients classified as either “unknown” or “undetermined” were excluded from formal data analysis. Statistical significance was set to *P* < 0.05.

## Results

### Characteristics of pediatric ocular trauma from all intentions

During the study period (2008–2014), 58,765 pediatric patients were admitted for trauma and had concomitant ocular injuries with a mean age (SD) of 11.9(6.9) years. Intentions, in descending order, were documented as unintentional (76.3%), assault (16.3%) and self-inflicted (0.7%). 6.7% injuries were of unknown or undetermined intent. For injuries of all intentions, most patients were male (68.7%), of White race (59.1%) and from the South (39.3%) and Midwest regions (22%). Blacks (17.6%), Hispanics (16.3%) and “others” (23.3%) represented smaller groups. Common locations were the street (42.1%) and home (29.3%) and common mechanisms were motor vehicle trauma-occupant (MVTO) (28.1%), struck-by-against (SBA) (16.1%) and falls (9.1%). When all vehicular accidents were totaled, they accounted for 43.9% of all injuries. Frequent injuries were contusions of eye/adnexa (30.6%), orbital injuries (29.9%), open wounds of ocular adnexa (29%) and superficial injuries (12.5%). Open globe injuries occurred in only 11.6% of patients. Mean (SD) ISS was 11.8(10.9). Traumatic brain injury was documented in 54.7% of patients. Mean (SD) hospital stay was 5(9) days and overall mortality rate was 2.9%.

### Unintentional trauma

**Fig. 1:**
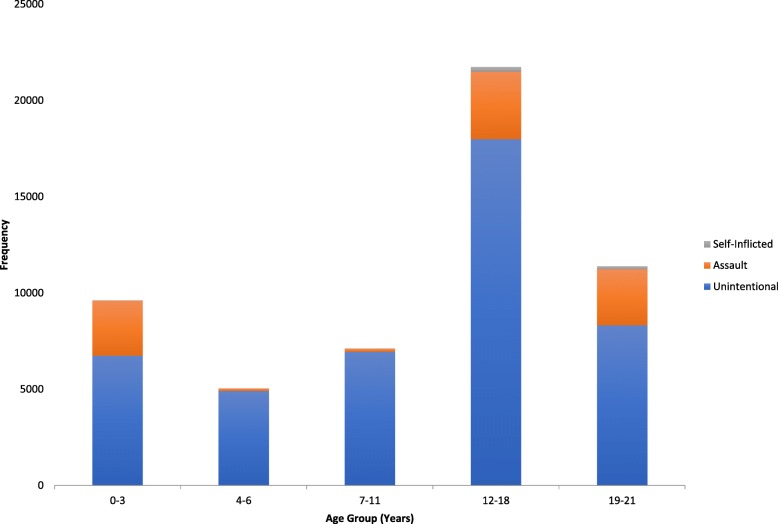
Frequency of Ocular Injuries in Different Age Groups Based Upon Intent

This group represented the most common intention of injury. The demographic breakdowns are described in Table 1. This group was predominately male (66.5%) and most commonly in the 12-18 year age group (40.1%) (Fig. 1). White race and the 7-11 year age group were at the highest risk of this intention. The most common location was the street (50.6%) and mechanism MVTO (36.0). Only 36.8% of MVTO patients were wearing seat belts at the time of injury. Most injuries were blunt (82.9%) and the most common injuries overall were open wound of the ocular adnexa (33.0%) and orbital injuries (29.7%). Open globe injuries occurred in only 11.1% of patients. Mean (SD) was ISS 11.7(11.1) and GCS was 13.1(3.9), representing mild to moderate injury severity. TBI occurred in 53.2% and the mortality rate was 2% (Table 1).

**Table 1:** Description of Findings in Ocular Injuries Secondary to Unintentional Intent in Pediatric Trauma, National Trauma Data Bank (2008-2014)

Characteristic	Number	Percentage (%)	Characteristic	Number	Percentage (%)	Mean (SD)	Median (IQR)
Year			Age			12.1 (6.4)	14 (6-18)
2008	5,893	13.2	0-3	6,739	15.0		
2009	6,289	14.0	4-6	4,919	11.0		
2010	6,428	14.3	7-11	6,945	15.5		
2011	6,366	14.2	12-18	17,979	40.1		
2012	6,822	15.2	19-21	8,230	18.4		
2013	6,434	14.4					
2014	6,580	14.7					
			Injury Severity Score			11.7(11.1)	9(4-17)
Gender			≤15	39,922	69.0		
Male	29,799	66.5	16-25	6,805	15.2		
Female	15,013	33.5	>25	5,312	11.9		
			Unknown	1773	4.0		
Race							
Black	6,498	14.5	GCS			13.1 (3.9)	15(14-15)
White	28,354	63.3	≤8	5,817	13.0		
Other	9,960	22.8	9-12	1,487	3.3		
			13-15	32,840	73.3		
Ethnicity			Unknown	4,668	10.4		
Hispanic	6,869	15.3					
Injury Type			Common Injuries				
Penetrating	1,893	4.2	Contusion cyc/adncxa	12,126	27.1		
Blunt	37,168	82.9	Orbital	13,308	29.7		
Other	5,751	12.9	Open adnexa wound	14,793	33.0		
			Superficial	5,768	12.9		
Traumatic Brain	23,834	53.2	Open wound eyeball	4,982	11.1		
Mortality	884	2.0	Visual pathway	590	1.3		
			Related cranial nerves	1284	2.9

### Assault-related trauma

This second intention of injury was similarly predominately male (77.5%), White (41.7%) and occured most frequently in the 12-18 year age group (36.6%) (Fig. 1). The full demographic information is provided in Table 2. Female victims of assault were slightly younger, (7.8+/− 8.5 years) than male victims (13.6+/− 7.5 years); *p* < 0.001 . Most injuries were blunt (48.2). Common ocular injuries were contusion of the eye and adnexa (46.3%), orbital injury (33.1%), and open globe injuries (10.8%). Frequent identifiable mechanisms were SBA (47.8%), firearms (13%) and falls (13%). Like unintentional injuries, ISS and GCS were clustered in the mild to moderate spectrum. TBI occurred in 63.1% and mortality rate was 6.6%.

**Table 2:** Description of Findings in Ocular Injuries Secondary to Assault in Pediatric Trauma, National Trauma Data Bank (2008-2014)

Characteristic	Number	Percentage (%)	Characteristic	Number	Percentage (%)	Mean (SD)	Median (IQR)
Year			Age			12.3(8.1)	17(1-19)
2008	1,323	13.8	0-3	2,872	30.0		
2009	1,486	15.5	4-6	132	1.4		
2010	1,428	14.9	7-11	157	1.6		
2011	1.385	14.5	12-18	3,505	36.6		
2012	1,393	14.5	19-21	2,913	30.4		
2013	1,271	13.3					
2014	1,293	13.5					
			Injury Severity Score			12.5(10.2)	9(5-18)
Gender			≤15	6,201	64.7		
Male	7,424	77.5	16-25	1,455	15.2		
Female	2,152	22.5	>25	1,503	15.7		
			Unknown	420	4.4		
Race							
Black	3,008	31.4					
White	3,996	41.7	CCS			12.7(4.4)	15(13-15)
Other	2,575	26.9	≤8	1,511	15.8		
			9-12	402	4.2		
Ethnicity			13-15	6,417	67.0		
Hispanic	1,953	20.4	Unknown	1,249	13.0		
Injury Type			Common Injuries				
Penetrating	1,241	13.0	Contusion eye/adnexa	4,432	46.3		
Blunt	4,614	48.2	Orbital	3,170	33.1		
Other	3,724	3,724	Open adnexa wound	1,349	14.1		
			Superficial	970	10.1		
Traumatic Brain Injury	6,049	63.1	Open wound eyeball	1,033	10.8		
Mortality	633	6.6	Visual pathway	258	2.7		
			Related cranial nerves	484	5.0		

*SD* Standard deviation, *IQR* Interquartile range, *GCS* Glasgow Coma Score

### Self-inflicted trauma

This group was by far the least common intention for pediatric ocular trauma. 80.5% were male, 68% white and 93.6%, between the ages of 12 and 21 years (Fig. 1). Table 3 describes the complete demographic breakdown of patients. The mean (SD) age was 17(3.4) years with no age disparity between males and females. The most common mechanism was firearms (59.0%) and location home (53.5%). Most injuries were penetrating (59.0%) and common injuries were orbital (42.6%), and open globe wounds (20.8%). The mean (SD) GCS was 8.3(5.4) indicating TBI of high severity. Likewise mean (SD) ISS was 21.0(11.8). TBI occurred in 78% and mortality rate was 19.9%. A detailed look at mechanisms in the seven (0-3 year) infants documented with self-inflicted injuries revealed they result from fall (1), firearms (5) and unspecified (1). Each survived their trauma.

**Table 3:** Description of Findings in Ocular Injuries Secondary to Self-Inflicted Intent in Pediatric Trauma, National Trauma Data Bank (2008-2014)

Characteristic	Number	Percentage (%)	Characteristic	Number	Percentage (%)	Mean (SD)	Median (IQR)
Year			Age			17.0 (3.4)	18.0(16-19)
2008	45	10.3	0-3	7	1.6		
2009	51	11.7	4-6	5	1.1		
2010	58	13.3	7-11	16	3.7		
2011	67	15.3	12-18	249	57.0		
2012	72	16.5	19-21	160	36.6		
2013	63	14.4					
2014	81	18.5					
			Injury Severity Score			21.0 (11.8)	22.0 (13-29)
Gender			≤l5	137	31.3		
Male	352	80.5	16-25	93	21.3		
Female	85	19.5	>25	194	44.4		
			Unknown	13	3.0		
Race							
Black	51	11.7					
White	297	68	GCS			8.3 (5.4)	6.0 (3-15)
Other	89	20.4	≤8	223	51.0		
			9-12	26	5.9		
Ethnicity			13-15	155	35.5		
Hispanic	56	12.8	Unknown	33	7.6		
Injury Type			Common Injuries				
Penetrating	258	59.0	Contusion eye/adnexa	115	26.3		
Blunt	95	21.7	Orbital	186	42.6		
Other	78	17.8	Open adnexa wound	66	15.1		
			Superficial	43	9.8		
Traumatic Brain Injury	341	78.0	Open wound eyeball	91	20.8		
Mortality	87	19.9	Visual pathway	45	10.3		
			Related cranial nerves	13	3.0		

*SD* Standard deviation, *IQR* Interquartile range, *GCS* Glasgow Coma Score

### Comparative analysis

#### Race, age and injuries

Unintentional injuries had greatest odds of occurring in the elementary school ages of 7-11 years (OR = 9.17, *p* < 0.001) and kindergarten ages of 4-6 years (OR = 7.73, *p* < 0.001). Assault, on the other hand, displayed a bimodal pattern and the 0-3 year group (OR = 2.38, *p* < 0.001) and the 19-21 year group (OR = 1.93. *p* < 0.001) had the greatest odds of assault. Self-inflicted injuries were most likely to occur in older patients in the 19-21 years (OR = 2.25, *p* < 0.001) and 12-18 years (OR = 2.04, *p* < 0.001) groups. The 0-3 year group were most likely to be injured at home (OR = 9.92, *p* < 0.001) and the oldest 19-21 year group, on the street(OR = 2.43, *p* < 0.001).

**Fig. 2:**
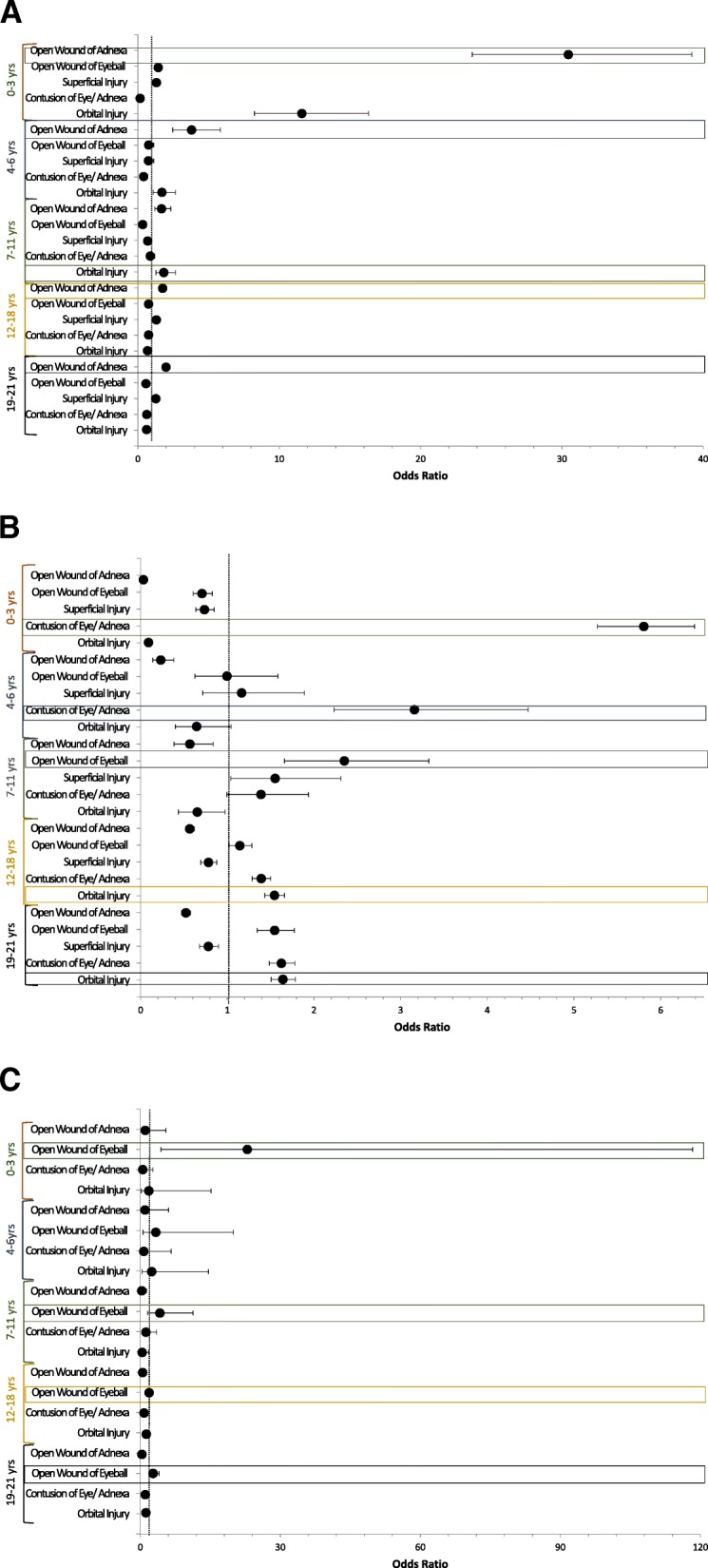
Summary of Multivariate Logistic Regression Analysis of Age Association with Type of Ocular Injury in Pediatric Patients with Ocular Trauma : **a** Summary of multivariate logistic regression with odds ratio and 95% confidence intervals analysis of age association with type of ocular injury in unintentional pediatric ocular trauma. Patients 0–3 years of age had 30.45 greater odds of open ocular adnexal wounds (*p* < 0.001) and a 11.60 odds of orbital injury (*p* < 0.001). Patients 4–6 years of age (OR = 3.80, *p* < 0.001), 12–18 years (OR = 1.75, *p* < 0.001) and 19–21 years (OR = 1.99, *p* < 0.001) had highest odds of open wounds of the ocular adnexa; those 7–11 years of age had 1.83 odds of orbital injuries (*p* = 0.001). **b** Summary of multivariate logistic regression with odds ratio and 95% confidence intervals analysis of age association with type of ocular injury in pediatric ocular trauma secondary to assault. 0–3 years (OR = 5.81, *p* < 0.001) and 4–6 years of age (OR = 3.16, *p* < 0.001) had highest odds of contusion of the eye/ adnexa; 7–11 years of open wounds of the eyeball (OR = 2.35, *p* < 0.001); and 12–18 years (OR = 1.54, *p* < 0.001) and 19–21 years (OR = 1.64, *p* = 0.001) of orbital injuries. **c** : Summary of multivariate logistic regression with odds ratio and 95% confidence intervals analysis of age association with type of ocular injury in self-inflicted pediatric ocular trauma. All age groups had increased odds of open wound injuries of the eyeball, with patients 0–3 years of age with 22.92 odds (*p* < 0.001); 7–11 years of age with 4.19 odds (*p* = 0.007); 12–18 years with 1.88 odds (*p* < 0.001) and 19–21 years with 2.74 odds (*p* < 0.001). Patients 4–6 years of age did not have statistically increased odds of the above five injury types

With respect to injuries, orbital injuries were most likely to be unintentional in the 0–3- and 4-6 year age groups while cranial nerve injuries were more likely to be unintentional in all groups except the 0-3 year group (Fig. 2 and Additional file 1: Table S1A). Assault injuries were more likely to result in ruptured globes in the 7-11 year group and injury to the optic nerve and pathways in the 0–3, 4–6 and 7-11 year age groups. (Fig. 2 and Additional file 1: Table S1B). Finally, self-inflicted injury was most strongly associated with injury to the optic nerve and pathways in the 12–18 and 19-21 year groups and ruptured globes in all age groups (Fig. 2 and Additional file 1: Table S1C).

**Fig. 3:**
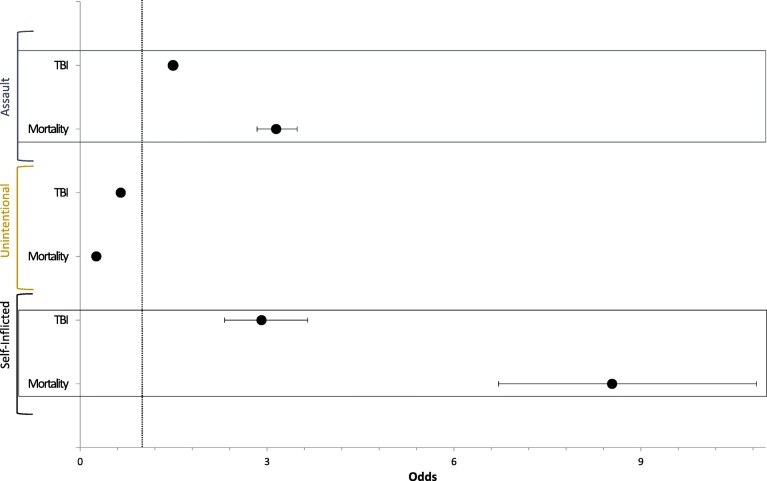
Summary of Simple Logistic Regression Analysis of Traumatic Brain Injury (TBI) and Mortality Associated with Intent of Injury in Pediatric Patients Ocular Trauma. Summary of simple logistic regression with odds ratio and 95% confidence intervals analysis of TBI and Mortality association with intent of injury in pediatric ocular trauma. TBI = Traumatic Brain Injury. Self-inflicted intent had the highest odds of TBI (OR = 2.91, *p* < 0.001) and mortality (OR = 8.54; *p* < 0.001), followed by assault injury with 1.49 odds of TBI (*p* < 0.001) and 3.15 odds of mortality (*p* < 0.001)

Unintentional (OR = 2.28, *p* < 0.001) and self-inflicted (OR = 1.45, *p* < 0.001) trauma were most associated with the White race and assault with the Black race (OR = 2.68, *p* < 0.001) and Hispanic ethnicity (OR = 1.41, *p* < 0.001). With respect to mechanisms, unintentional SBA had greater odds of occurring in the 0-3 year group in Blacks (OR = 1.81, *p* < 0.001) and Hispanics (OR = 1.62, *p* < 0.001) but in the 12-18 years group in Whites (OR = 1.59, *p* < 0.001). This pattern was repeated for MVTO, with greatest odds of causing injuries in the youngest age group in both Blacks (OR = 1.93, *p* < 0.001) and Hispanics (OR = 1.65, *p* < 0.001) and an older demographic in Whites (OR = 1.4. *p* < 0.001); likewise, for falls with the 0-3 year group for Blacks (OR = 1.54, *p* < 0.001), Hispanics (OR = 1.43, *p* < 0.001) and the oldest group for Whites (OR = 1.62, *p* < 0.001). Assault-related SBA displayed a propensity to older groups: 7-11 year for Blacks (OR = 1.84, *p* = 0.01), 12-18 year for Hispanics (OR = 1.18, *p* = 0.02) and 19-21 year group for Whites (OR = 1.44, p < 0.001). No statistically significant race or ethnic variances between age groups were noted for firearms with respect to intent (Fig. 3 and Additional file 1: Table S2).

#### Trauma severity

There was a tendency towards greater injury severity, more association with TBI, longer hospital stays and mortality from unintentional to assault and self-inflicted trauma. ISS for unintentional trauma was mostly of minor severity (ISS = 1–8) (OR = 1.33, *p* < 0.001) while assault (OR = 1.29, *p* < 0.001) and self-inflicted trauma (OR = 5.39, *p* < 0.001) had greatest odds of the very severe designation (ISS > 24). Similarly, Self-inflicted trauma had the greatest odds of association with TBI (OR = 2.91, *p* < 0.001). Unintentional trauma had highest odds of mild to moderate TBI (GCS = 13–15) (OR = 1.51, *p* < 0.001), while assault had highest odd of moderate TBI (GCS = 9–12) (OR = 1.29, *p* < 0.001) and self-inflicted injury, severe TBI (GCS < 8) (OR = 6.87, *p* < 0.001). Consequently, mortality was most associated with self-inflicted trauma (OR = 8.54, *p* < 0.001) followed by assault (OR = 3.15, *p* < 0.001). Assault was most likely to lead to death in the 0-3 year group (OR = 8.52, p < 0.001) and unintentional injury in the 19-21 year group (OR = 2.08, *p* < 0.001). There was no significant age disparity in mortality from self-inflicted trauma.

## Discussion

In this study of the NTDB (2008–2014), our results reaffirm findings of previous trauma reports. There was an increasing trend in age from unintentional to assault and self-inflicted trauma with a corresponding increase in injury severity, association with and degree of traumatic brain injury (TBI), hospital stay and mortality rate (Tables 1, 2 and 3). There were strong associations between types of injuries and age groups based on intention with the younger ages suffering sight-threatening injuries, including open adnexa wounds, open globe, orbital and optic nerves and visual pathways injuries from both unintentional trauma and assault injuries while older groups had sight-threatening injuries mostly following assault-related injuries. Self-inflicted trauma showed greater association with open globe and optic nerve and visual pathway injuries in the youngest and oldest age groups respectively. There was further disparity between race and ethnicity with Whites more likely to suffer unintentional and self-inflicted trauma and Blacks and Hispanics, assault-related trauma.

Most studies addressing intention in pediatric trauma have focused on NAT in infants [12–14]. Several studies had focused on intention of trauma in broader pediatric ages [28–31]. In a recent study of children between 0 and 19 years, Ballesteros et al. demonstrated similar findings to our study with male and adolescent predominance and unintentional trauma outnumbering assault and self-inflicted trauma [28]. Also, they noted that the youngest children were at greatest risk of succumbing to their injuries. Blacks and Hispanics were disproportionately represented in the assault group and whites in the self-inflicted group [28]. Considering all races/ethnicities, they demonstrated a bimodal age distribution for all intentions that, in our study, was most obvious only in the assault group. These differences might have resulted from surveying different databases, over different time periods. Our data also included the 19-21 year group and had lower proportions of American Indians and Alaskan Natives. However, variance between the common demographic groups we both surveyed were borne out. Other studies who noted this race/ethnic disparity have attributed these differences to socioeconomic constraints, exposure to crime and/or suicide and limited resources [32, 33].

Barampas et al. utilized the NTDB (2007–2011) to examine pediatric assault-related injuries [3]. Similarly, they found male predominance and disproportionate Black victims but also noted infants having the highest association with TBI. Although the rate of assault was slightly lower than in this study (16% vs 10%), their mortality rate of 8% was similar to our assault cohort [3]. Estroff et al. compared the epidemiology of accidental and non-accidental trauma (NAT) over four years and found that victims of NAT had a significantly higher mortality rate and injury severity [16]. These findings comport with the increased injury severity, TBI and mortality rate in the 0-3 year age group following assault injuries in our study.

While ocular injuries commonly occur in adult and pediatric patients admitted with major trauma few have reported the epidemiology of pediatric ocular trauma [34–37]. Guly et al., to our knowledge, conducted the only study of ocular injuries in major trauma that included intent in its analysis [37]. They studied patients of all ages and noted that assault resulted in 10.5% and “non-accidental injury” in 4.4% which together were slightly lower than the 16.3% we found [37]. They did not comment on the risk of mortality nor associate demographic groups, types of injury and mechanisms with intent.

Garcia et al. performed the only studies, to date, of pediatric ocular injury in major trauma [35, 36]. One used the National Pediatric Trauma Registry to study 7497 patients and demonstrated strong male predominance with over 70% being victims of various vehicular accidents. This was greater than the proportion of all vehicular accidents (43.9%) in this study [35]. Again, these disparities likely reflect the different databases sourced. Our study, additionally, showed variances in trauma severity and association with TBI. Assault was associated with the highest risk of TBI and mortality in the 0-3 year group while self-inflicted trauma had the highest risk of TBI and mortality in older children. This was expected since the most common mechanism was firearms. Although there was a lower injury severity and rate of TBI, unintentional trauma was most associated with mortality in the 19-21 year group. This may have resulted from a combined MVTO and other vehicular injuries accounting for the largest group of mechanisms overall. An important finding in this study, is that amongst victims of MVTO, an alarmingly low 36.8% were wearing restraints. It has been shown that non-compliance with seatbelts leads to increased injury severity and mortality and longer hospitalizations [38–40]. Clearly, compliance with and enforcement of seatbelt laws is one avenue of intervention aimed at reducing pediatric ocular trauma and mortality.

### Study limitations

This study had several limitations. Firstly, it was a retrospective survey of a database with multiple sources. NTDB submissions are based on data collected by Emergency Department and Trauma services and may underestimate ophthalmic injury. Also, circumstances surrounding each trauma were not available and could have clarified or helped to re-define the inexplicable self-inflicted injuries designation in the 0-3 year group. Secondly, the ICD-9CM used in this period lacked the diagnostic precision of the current ICD-10CM codes. Lastly, the results were likely skewed towards more severe injuries since all patients were admitted.

## Conclusions

This study demonstrated the impact of intention of trauma on types of pediatric ocular injury, injury severity, associated TBI and mortality. While affirming previous findings of male predominance and gender and racial/ethnic disparities, we noted that visually-threatening injuries occurred in both assault and unintentional trauma in the younger children while being more common in assault and self-inflicted injuries in older age groups. Most patients in all trauma intentions groups survived their injuries and greater injury severity and TBI were most common in infants and associated with assault and self-inflicted trauma.

## Additional file

Additional file 1: **Table S1A.** Summary of Regression Analysis of Ocular Injury vs Age in Pediatric Ocular Trauma Secondary to Unintentional Injury. **Table S1B.** Summary of Regression Analysis of Ocular Injury Vs Age in Pediatric Ocular Trauma Secondary to Assault. **Table S1C.** Summary of Regression Analysis of Ocular Injury Vs Age in Pediatric Ocular Trauma Secondary to Self-inflicted Injury. **Table S2.** Summary of Regression analysis of Race and Ethnicity vs Intent in Pediatric Ocular Trauma. (DOCX 43 kb)

## Abbreviations

CDC: Center for disease control
CI: Confidence interval
GCS: Glasgow coma scale
ICD-9CM: International classification of diseases, 9th revision, clinical modification
IQR: Interquartile range
ISS: Injury severity score
MVTO: Motor vehicle trauma occupant
NAT: Non-accidental trauma
NTDB: National trauma data bank
OR: Odds ratio
SBA: Struck-by-against
SD: Standard deviation
SPSS: Statistical package for science
TBI: Traumatic brain injury
