# Supplementary material for: A comparison of pediatric ocular injuries based on intention in patients admitted with trauma
Source: BMC Ophthalmol. 2019 Jan 29;19:37. doi: 10.1186/s12886-018-1024-7 (PMC6352334; doi:10.1186/s12886-018-1024-7)
Supplement: Supplementary file 1 — Table S1A. Summary of Regression Analysis of Ocular Injury vs Age in Pediatric Ocular Trauma Secondary to Unintentional Injury. Table S1B. Summary of Regression Analysis of Ocular Injury Vs Age in Pediatric Ocular Trauma Secondary to Assault. Table S1C. Summary of Regression Analysis of Ocular Injury Vs Age in Pediatric Ocular Trauma Secondary to Self-inflicted Injury. Table S2. Summary of Regression analysis of Race and Ethnicity vs Intent in Pediatric Ocular Trauma. (DOCX 43 kb) [file 12886_2018_1024_MOESM1_ESM.docx]

**Additional file 1: Table S1A:** Logistic Regression Analysis of Ocular Injury Vs Age in Pediatric Ocular Trauma Secondary to Unintentional Injury

|  |  |  |  |  |  |
| --- | --- | --- | --- | --- | --- |
| **Ocular Injury** | **Age Group (Total Frequency)** | **Frequency (%)** | **p-value** | **Odds Ratio** | **95% Confidence Interval** |
| Open wound of ocular adnexa | 0-3 (n=6739) | 2639 (39.2) | <0.001 | 30.453 | 23.661-39.195 |
|  | 4-6 (n=4919) | 1996 (40.6) | <0.001 | 3.797 | 2.471-5.834 |
|  | 7-11 (n=6945) | 2220 (32.0) | 0.002 | 1.671 | 1.196-2.334 |
|  | 12-18 (n=17979) | 5227 (29.1) | <0.001 | 1.752 | 1.606-1.910 |
|  | 19-21  (n=8230) | 2711 (32.9) | <0.001 | 1.989 | 1.801-2.196 |
| Open wound of eye ball | 0-3 (n=6739) | 730 (10.8) | <0.001 | 1.437 | 1.234-1.674 |
|  | 4-6 (n=4919) | 816 (16.6) | 0.162 | 0.760 | 0.517-1.117 |
|  | 7-11 (n=6945) | 1052 (15.1) | <0.001 | 0.337 | 0.251-0.453 |
|  | 12-18 (n=17979) | 1770 (9.8) | <0.001 | 0.758 | 0.681-0.844 |
|  | 19-21  (n=8230) | 614 (7.5) | <0.001 | 0.570 | 0.498-0.652 |
| Superficial Injury | 0-3 (n=6739) | 848 (12.6) | <0.001 | 1.311 | 1.141-1.505 |
|  | 4-6 (n=4919) | 650 (13.2) | 0.153 | 0.740 | 0.488-1.120 |
|  | 7-11 (n=6945) | 950 (13.7) | 0.047 | 0.696 | 0.486-0.997 |
|  | 12-18 (n=17979) | 2339 (13) | <0.001 | 1.305 | 1.166-1.460 |
|  | 19-21  (n=8230) | 981 (11.9) | 0.001 | 1.267 | 1.106-1.452 |
| Contusion of Eye and Adnexa | 0-3 (n=6739) | 2007 (29.8) | <0.001 | 0.159 | 0.145-0.175 |
|  | 4-6 (n=4919) | 1212 (24.6) | <0.001 | 0.408 | 0.298-0.558 |
|  | 7-11 (n=6945) | 1909 (27.5) | 0.384 | 0.874 | 0.646-1.183 |
|  | 12-18 (n=17979) | 4952 (27.5) | <0.001 | 0.756 | 0.701-0.814 |
|  | 19-21  (n=8230) | 2046 (24.9) | <0.001 | 0.630 | 0.576-0.688 |
| Foreign Body on External Eye | 0-3 (n=6739) | 8 (0.1) | 0.734 | 1.836 | 0.390-8.652 |
|  | 4-6 (n=4919) | 13 (0.3) | 1.000 | - | - |
|  | 7-11 (n=6945) | 21 (0.3) | 0.140 | 0.308 | 0.072-1.322 |
|  | 12-18 (n=17979) | 79 (0.4) | <0.010 | 2.835 | 1.235-6.506 |
|  | 19-21  (n=8230) | 42 (0.5) | 0.002 | 5.347 | 1.656-17.262 |
| Burn Confined to Eye and Adnexa | 0-3 (n=6739) | 393 (5.8) | <0.001 | 8.642 | 5.613-13.306 |
|  | 4-6 (n=4919) | 86 (1.7) | 0.076 | 0.469 | 0.202-1.088 |
|  | 7-11 (n=6945) | 103 (1.5) | 0.551 | 0.756 | 0.276-2.074 |
|  | 12-18 (n=17979) | 151 (0.8) | <0.001 | 3.261 | 1.718-6.190 |
|  | 19-21  (n=8230) | 65 (0.8) | 0.001 | 3.107 | 1.489-6.482 |
| Orbital injury (no other facial fracture) | 0-3 (n=6739) | 790 (11.7) | <0.001 | 11.599 | 8.244-16.318 |
|  | 4-6 (n=4919) | 1069 (21.7) | 0.018 | 1.702 | 1.090-2.659 |
|  | 7-11 (n=6945) | 1853 (26.7) | 0.001 | 1.830 | 1.282-2.655 |
|  | 12-18 (n=17979) | 6501 (36.2) | <0.001 | 0.677 | 0.631-0.727 |
|  | 19-21  (n=8230) | 3095 (37.6) | <0.001 | 0.618 | 0.568-0.671 |
| Injury to Optic Nerve and pathways | 0-3 (n=6739) | 80 (1.2) | <0.001 | 0.263 | 0.199-0.348 |
|  | 4-6 (n=4919) | 41 (0.8) | 0.015 | 0.267 | 0.104-0.685 |
|  | 7-11 (n=6945) | 91 (1.3) | 0.007 | 0.327 | 0.157-0.683 |
|  | 12-18 (n=17979) | 273 (1.5) | <0.001 | 0.546 | 0.435-0.685 |
|  | 19-21  (n=8230) | 105 (1.3) | 0.002 | 0.619 | 0.452-0.847 |
| Injury to other cranial nerves | 0-3 (n=6739) | 160 (2.4) | <0.001 | 0.199 | 0.164-0.241 |
|  | 4-6 (n=4919) | 140 (2.8) | 0.630 | 1.572 | 0.496-4.987 |
|  | 7-11 (n=6945) | 209 (3.0) | 0.380 | 1.559 | 0.574-4.235 |
|  | 12-18 (n=17979) | 557 (3.1) | 0.065 | 1.227 | 0.987-1.526 |
|  | 19-21  (n=8230) | 218 (2.6) | 0.040 | 1.346 | 1.012-1.791 |

**Table 1B:** Logistic Regression Analysis of Ocular Injury versus Age secondary to Assault

| **Ocular Injury** | **Age Group (Total Frequency)** | **Frequency (%)** | **p-value** | **Odds Ratio** | **95% Confidence Interval** |
| --- | --- | --- | --- | --- | --- |
| Open wound of ocular adnexa | 0-3 (n=2872) | 56 (1.9) | <0.001 | 0.032 | 0.025-0.042 |
|  | 4-6 (n=132) | 18 (13.6) | <0.001 | 0.232 | 0.141-0.383 |
|  | 7-11 (n=157) | 33 (21.0) | 0.004 | 0.568 | 0.385-0.836 |
|  | 12-18 (n=3505) | 657 (18.7) | <0.001 | 0.567 | 0.518-0.621 |
|  | 19-21  (n=2913) | 585 (20.1) | <0.001 | 0.522 | 0.471-0.577 |
| Open wound of eye ball | 0-3 (n=2872) | 225 (7.8) | <0.001 | 0.708 | 0.606-0.827 |
|  | 4-6 (n=132) | 22 (16.7) | 0.986 | 0.996 | 0.626-1.584 |
|  | 7-11 (n=157) | 47 (29.9) | <0.001 | 2.35 | 1.660-3.326 |
|  | 12-18 (n=3505) | 400 (11.4) | 0.22 | 1.144 | 1.020-1.283 |
|  | 19-21  (n=2913) | 339 (11.6) | <0.001 | 1.546 | 1.347-1.775 |
| Superficial Injury | 0-3 (n=2872) | 276 (9.6) | <0.001 | 0.736 | 0.638-0.849 |
|  | 4-6 (n=132) | 20 (15.2) | 0.535 | 1.165 | 0.719-1.888 |
|  | 7-11 (n=157) | 31 (19.7) | 0.03 | 1.552 | 1.041-2.312 |
|  | 12-18 (n=3505) | 365 (10.4) | <0.001 | 0.783 | 0.696-0.879 |
|  | 19-21  (n=2913) | 278 (9.5) | 0.001 | 0.781 | 0.679-0.899 |
| Contusion of Eye and Adnexa | 0-3 (n=2872) | 2082 (72.5) | <0.001 | 5.805 | 5.271-6.391 |
|  | 4-6 (n=132) | 67 (50.8) | <0.001 | 3.159 | 2.232-4.471 |
|  | 7-11 (n=157) | 54 (34.4) | 0.053 | 1.388 | 0.995-1.938 |
|  | 12-18 (n=3505) | 1209 (34.5) | <0.001 | 1.391 | 1.288-1.502 |
|  | 19-21  (n=2913) | 1020 (35.0) | <0.001 | 1.624 | 1.484-1.779 |
| Foreign Body on External Eye | 0-3 (n=2872) | 1 (0.0) | 0.299 | 0.269 | 0.034-2.124 |
|  | 4-6 (n=132) | 0 (0.0) | - | - | - |
|  | 7-11 (n=157) | 1 (0.6) | 0.4 | 2.031 | 0.272-15.164 |
|  | 12-18 (n=3505) | 4 (0.1) | 0.004 | 0.257 | 0.094-0.703 |
|  | 19-21  (n=2913) | 3 (0.1) | <0.003 | 0.206 | 0.064-0.666 |
| Burn Confined to Eye and Adnexa | 0-3 (n=2872) | 14 (0.5) | <0.001 | 0.08 | 0.047-0.137 |
|  | 4-6 (n=132) | 4 (3.0) | 0.303 | 1.727 | 0.624-4.776 |
|  | 7-11 (n=157) | 3 (1.9) | 0.51 | 1.29 | 0.405-4.112 |
|  | 12-18 (n=3505) | 5 (0.1) | <0.001 | 0.166 | 0.068-0.406 |
|  | 19-21  (n=2913) | 5 (0.2) | <0.001 | 0.212 | 0.085-0.526 |
| Orbital injury (no other facial fracture) | 0-3 (n=2872) | 33 (1.1) | <0.001 | 0.091 | 0.064-0.129 |
|  | 4-6 (n=132) | 20 (15.2) | 0.073 | 0.646 | 0.400-1.045 |
|  | 7-11 (n=157) | 30 (19.1) | 0.036 | 0.653 | 0.437-0.976 |
|  | 12-18 (n=3505) | 1634 (46.6) | <0.001 | 1.544 | 1.435-1.660 |
|  | 19-21  (n=2913) | 1453 (49.9) | 0.001 | 1.642 | 1.508-1.787 |
| Injury to Optic Nerve and pathways | 0-3 (n=2872) | 132 (4.6) | <0.001 | 3.991 | 3.022-5.271 |
|  | 4-6 (n=132) | 4 (3.0) | 0.031 | 3.653 | 1.290-10.339 |
|  | 7-11 (n=157) | 6 (3.8) | 0.021 | 2.948 | 1.271-6.837 |
|  | 12-18 (n=3505) | 71 (2.0) | 0.151 | 1.21 | 0.932-1.570 |
|  | 19-21  (n=2913) | 45 (1.5) | 0.768 | 1.053 | 0.747-1.485 |
| Injury to other cranial nerves | 0-3 (n=2872) | 334 (11.6) | <0.001 | 5.487 | 4.525-6.653 |
|  | 4-6 (n=132) | 2 (1.5) | 0.589 | 0.525 | 0.129-2.142 |
|  | 7-11 (n=157) | 4 (2.5) | 1.000 | 0.849 | 0.311-2.312 |
|  | 12-18 (n=3505) | 86 (2.5) | 0.039 | 0.785 | 0.624-0.988 |
|  | 19-21  (n=2913) | 58 (2.0) | 0.056 | 0.753 | 0.562-1.008 |

**Table 1C:** Logistic Regression Analysis of Ocular Injury versus Age Secondary to Self-Inflicted Trauma

| **Ocular Injury** | **Age Group (Total Frequency)** | **Frequency (%)** | **p-value** | **Odds Ratio** | **95% Confidence Interval** |
| --- | --- | --- | --- | --- | --- |
| Open wound of ocular adnexa | 0-3 (n=7) | 2 (28.6) | 1.000 | 1.055 | 0.205-5.440 |
|  | 4-6 (n=5) | 2 (40.0) | 1.000 | 1.010 | 0.169-6.050 |
|  | 7-11 (n=16) | 2 (12.5) | 0.099 | 0.307 | 0.070-1.354 |
|  | 12-18 (n=249) | 40 (16.1 ) | <0.001 | 0.507 | 0.361-0.712 |
|  | 19-21  (n=160) | 20 (12.5) | <0.001 | 0.340 | 0.213-0.545 |
| Open wound of eye ball | 0-3 (n=7) | 5 (71.4) | <0.001 | 22.924 | 4.442-118.317 |
|  | 4-6 (n=5) | 2 (40.0) | 0.197 | 3.325 | 0.555-19.932 |
|  | 7-11 (n=16) | 7 (43.8) | 0.007 | 4.194 | 1.559-11.285 |
|  | 12-18 (n=249) | 44 (17.7) | <0.001 | 1.880 | 1.354-2.612 |
|  | 19-21  (n=160) | 33 (20.6) | <0.001 | 2.743 | 1.860-4.046 |
| Superficial Injury | 0-3 (n=7) | 0 (0.0) | - | - | - |
|  | 4-6 (n=5) | 0 (0.0) | - | - | - |
|  | 7-11 (n=16) | 3 (18.8) | 0.477 | 1.441 | 0.410-5.064 |
|  | 12-18 (n=249) | 21 (8.4) | 0.050 | 0.640 | 0.409-1.003 |
|  | 19-21  (n=160) | 19 (11.9) | 0.815 | 1.059 | 0.654-1.716 |
| Contusion of Eye and Adnexa | 0-3 (n=7) | 2 (28.6) | 0.706 | 0.524 | 0.102-2.702 |
|  | 4-6 (n=5) | 1 (20) | 1.000 | 0.739 | 0.082-6.615 |
|  | 7-11 (n=16) | 5 (31.3) | 0.781 | 1.195 | 0.415-3.443 |
|  | 12-18 (n=249) | 61 (24.5) | 0.151 | 0.809 | 0.605-1.081 |
|  | 19-21  (n=160) | 46 (28.7) | 0.721 | 1.065 | 0.754-1.503 |
| Foreign Body on External Eye | 0-3 (n=7) | 0 (0.0) | - | - | - |
|  | 4-6 (n=5) | 0 (0.0) | - | - | - |
|  | 7-11 (n=16) | 0 (0.0) | - | - | - |
|  | 12-18 (n=249) | 1 (0.4) | 0.623 | 1.032 | 0.143-7.444 |
|  | 19-21  (n=160) | 0 (0.0) | - | - | - |
| Burn Confined to Eye and Adnexa | 0-3 (n=7) | 0 (0.0) | - | - | - |
|  | 4-6 (n=5) | 0 (0.0) | - | - | - |
|  | 7-11 (n=16) | 0 (0.0) | - | - | - |
|  | 12-18 (n=249) | 4 (1.6) | 0.113 | 2.229 | 0.820-6.061 |
|  | 19-21  (n=160) | 2 (1.3) | 0.275 | 1.984 | 0.482-8.160 |
| Orbital injury (no other facial fracture) | 0-3 (n=7) | 1 (14.3) | 0.459 | 1.820 | 0.219-15.139 |
|  | 4-6 (n=5) | 2 (40.0) | 0.293 | 2.439 | 0.407-14.616 |
|  | 7-11 (n=16) | 2 (12.5) | 0.266 | 0.398 | 0.090-1.752 |
|  | 12-18 (n=249) | 109 (43.8 ) | 0.051 | 1.284 | 0.998-1.652 |
|  | 19-21  (n=160) | 72 (45.0) | 0.283 | 1.187 | 0.868-1.625 |
| Injury to Optic Nerve and pathways | 0-3 (n=7) | 0 (0.0) | - | - | - |
|  | 4-6 (n=5) | 0 (0.0) | - | - | - |
|  | 7-11 (n=16) | 1 (6.3) | 0.200 | 4.786 | 0.626-36.591 |
|  | 12-18 (n=249) | 30 (12.0) | <0.001 | 8.337 | 5.611-12.388 |
|  | 19-21  (n=160) | 14 (8.8) | <0.001 | 6.833 | 3.861-12.092 |
| Injury to other cranial nerves | 0-3 (n=7) | 0 (0.0) | - | - | - |
|  | 4-6 (n=5) | 0 (0.0) | - | - | - |
|  | 7-11 (n=16) | 0 (0.0) | - | - | - |
|  | 12-18 (n=249) | 9 (3.6) | 0.567 | 1.216 | 0.622-2.376 |
|  | 19-21  (n=160) | 4 (2.5) | 0.800 | 1.015 | 0.373-2.758 |

**Table S2** - Logistic Regression Analysis of Race and Ethnicity vs Intent in Pediatric Ocular Trauma

| **Race/ Ethnicity (Total Frequency)** | **Intent** | **Frequency (%)** | **p-value** | **Odds Ratio** | **95% Confidence Interval** |
| --- | --- | --- | --- | --- | --- |
| White (n=32852) | Unintentional | 28354 (86.3) | <0.001 | 2.280 | 2.184-2.381 |
|  | Assault | 3996 (12.2) | <0.001 | 0.417 | 0.399-0.347 |
|  | Self-inflicted | 297 (0.9) | <0.001 | 1.451 | 1.186-1.776 |
| Black (n=9674) | Unintentional | 6498 (67.2) | <0.001 | 0.388 | 0.370-0.408 |
|  | Assault | 3008 (31.1) | <0.001 | 2.679 | 2.548-2.818 |
|  | Self-inflicted | 51 (0.5) | <0.001 | 0.621 | 0.463-0.832 |
| Hispanic (n=54828) | Unintentional | 6869 (15.3) | <0.001 | 0.724 | 0.686-0.765 |
|  | Assault | 1953 (20.4) | <0.001 | 1.414 | 1.337-1.495 |
|  | Self-inflicted | 56 (12.8) | 0.053 | 0.758 | 0.572-1.005 |
